# Ⅰ期肺腺癌VATS肺叶切除与亚肺叶切除预后比较

**DOI:** 10.3779/j.issn.1009-3419.2017.01.07

**Published:** 2017-01-20

**Authors:** 洋 刘, 声逸 钟, 绮华 何, 剑嵘 张, 学炜 陈, 敏章 郭, 建行 何

**Affiliations:** 1 510515 广州，南方医科大学研究生院 Southern Medical University, Guangzhou 510515, China; 2 510120 广州，广州医科大学附属第一医院胸外科 Department of Thoracic Surgery, the First Affiliated Hospital of Guangzhou Medical University, Guangzhou 510120, China

**Keywords:** VATS, 肺肿瘤, 亚肺叶切除, 肺叶切除, 预后, Video-assisted thoracic surgery, Lung neoplasms, VATS lobectomy, VATS sublobe resection, Prognosis

## Abstract

**背景与目的:**

美国国立综合癌症网络（National Comprehensive Cancer Network, NCCN）指南推荐，大部分可手术切除的肺癌首选电视辅助胸腔镜手术（video-assisted thoracoscopic surgery, VATS）解剖性肺叶切除。而研究证实肺段切除Ⅰ期肺癌对肺功能的保护优于肺叶切除。目前，临床上对Ⅰ期肺腺癌VATS亚肺叶切除能否获得与肺叶切除同等疗效仍未确定，现分析两种手术方式治疗Ⅰ期肺腺癌预后的比较。

**方法:**

回顾性研究2009年1月-2011年12月广州医科大学附属第一医院收治的Ⅰ期肺腺癌患者，其中VATS肺叶切除222例，亚肺叶切除36例；对两组患者使用倾向评分匹配（propensity score matching, PSM），比较两组患者的临床病理特征及生存预后。

**结果:**

两组匹配患者35例，匹配后VATS肺叶切除组与亚肺叶切除组的术后无病生存期（disease free survival, DFS）分别为49.3个月、42.7个月，差异无统计学意义（*P*=0.137）；两组术后总生存期（overall survival, OS）分别为50.3个月、49.0个月，差异无统计学意义（*P*=0.122）。分期分层结果示，Ia期肺叶切除和亚肺叶切除两组术后DFS差异无统计学意义；而Ib期肺叶切除和亚肺叶切除两组术后DFS差异有统计学意义。

**结论:**

Ia期肺腺癌VATS亚肺叶切除的生存预后不亚于肺叶切除，Ib期肺腺癌建议选择VATS肺叶切除治疗。

## Abstract (en)

**Background and objective:**

National Comprehensive Cancer Network (NCCN) guidelines recommend video-assisted thoracoscopic surgery (VATS) anatomical lobectomy as the first choice for the treatment of resectable lung cancer. However, sublobar resection offers significantly better functional preservation compared with lobectomy for stage Ⅰ lung cancer. At present, the inferiority of sublobar resection to lobectomy is still uncertain. Herein, we compared the prognoses of these two types of surgical treatment for stage Ⅰ lung adenocarcinoma.

**Methods:**

Retrospective research was conducted on 258 patients with stage Ⅰ lung adenocarcinomas who underwent VATS lobectomy and sublobar resection at the First Affiliated Hospital of Guangzhou Medical University between January 2009 and December 2011. VATS lobectomy was performed on 222 patients, and VATS sublobe resection was performed on 36 patients. Propensity score matching analyses were conducted on the two groups.

**Results:**

A total of 70 patients were matched in the two groups. No significant difference was observed between the lobectomy and sublobar resection groups after matching (*P*=0.137). The disease-free survival (DFS) of the two groups were 49.3 and 42.7 months, and their overall survival (OS) were 50.3 and 49.0 months (*P*=0.122). Further, stratified analysis showed no significant differences in DFS and OS between the two groups with stage Ia lung adenocarcinoma. Nevertheless, the DFS and OS of the two groups significantly differed in matched patients with stage Ib lung adenocarcinomas.

**Conclusion:**

Sublobar resection could achieve a similar prognosis to VATS lobectomy for stage Ia lung adenocarcinoma. Lobectomy should still be the first choice for the treatment of stage Ib lung adenocarcinoma.

目前，肺癌仍居我国肿瘤致死率的榜首^[1]^。1995年，肺癌研究组（Lung Cancer Study Group, LCSG）研究结果报道，外周型早期（T1N0）非小细胞肺癌（non-small cell lung cancer, NSCLC）局限性切除（122例）组术后死亡率和局部复发率高于肺叶切除（125例）^[2]^。最新美国国立综合癌症网络（National Comprehensive Cancer Network, NCCN）指南推荐，大部分可手术切除肺癌首选电视辅助胸腔镜手术（video-assisted thoracoscopic surgery, VATS）解剖性肺叶切除。然而有研究^[3, 4]^证实，Ⅰ期肺癌肺段切除对肺功能的保护优于肺叶切除。

肺腺癌随着诊出所占NSCLC比例不断攀升，已成为肺癌中最常见的组织学类型^[5]^。目前，临床外科医师对于Ⅰ期肺腺癌行VATS亚肺叶切除能否获得与肺叶切除同等疗效仍尚未形成共识^[6]^。我们现回顾分析广州医科大学附属第一医院收治的258例Ⅰ期肺腺癌的临床病理资料，对VATS肺叶切除与亚肺叶切除患者采用倾向评分匹配（propensity score matching, PSM）后进行生存预后分析比较，评估两种术式治疗的远期疗效，以期用于临床实践。

## 1 资料和方法

### 1.1 资料

回顾2009年1月1日-2011年12月31日在我院胸外科接受VATS手术治疗患者。纳入标准如下：术前评估排除纵隔或远处转移；接受VATS解剖性肺叶或亚肺叶（楔形或肺段）切除术治疗；术后病理为Ⅰ期肺腺癌患者[按照国际肺癌研究协会（International Association for the Study of Lung Cancer, IASLC）第7版TNM（Tumor Node Metastasis）分期标准，pT1-2aN0M0]。

本研究共纳入患者258例，其中男性127例，女性131例。中位年龄60（20-85）岁。157例因体检发现肺部肿块影，101例患者首诊主诉有咳嗽、咳血、胸痛、呼吸困难等症状。接受VATS解剖性肺叶切除222例；VATS亚肺叶切除患者36例（其中肺楔形切除16例，单肺段切除10例，双肺段切除4例，多肺段切除6例）。Ia期患者93例，Ib期患者165例。具体肿瘤部位见表 1。

**1:** Ⅰ期肺腺癌患者的临床病理特征 Clinical pathological characteristics of patients with stage Ⅰ lung adenocarcinoma

Variables	Surgical method		*P* value
	Lobectomy (*n*=222)	Sublobectomy (*n*=36)	
Gender			0.240
Male	106 (47.7%)	21 (58.3%)	
Female	116 (52.3%)	15 (41.7%)	
Age (yr)	60.32±10.81	61.53±13.25	0.549
BMI (kg/m^2^)	22.14±4.82	22.65±4.79	0.056
Symptom			0.688
No	134 (60.4%)	23 (63.9%)	
Yes	88 (39.6%)	13 (36.1%)	
Smoking status			0.327
Never	161 (72.5%)	23 (63.9%)	
Ever	55 (24.8%)	9 (25.0%)	
Unknown	6 (2.7%)	4 (11.1%)	
Tumor site			0.330
RUL	87 (39.2%)	13 (36.1%)	
RML	16 (7.2%)	0 (0.0%)	
RLL	41 (18.5%)	6 (16.7%)	
LUL	44 (19.8%)	12 (33.3%)	
LLL	34 (15.3%)	5 (13.9%)	
Tumor size (cm)	2.42±1.06	1.96±0.99	0.016
NLNS	5.07±1.45	3.28±1.67	＜0.001
VPI			0.438
No	91 (41.0%)	17 (47.2%)	
Yes	131 (59.1%)	19 (52.8%)	
BMI: body mass index; RUL: right upper lung; RML: right middle lobe; RLL: right lower lung; LUL: left upper lung; LLL: left lower lobe; VPI: visceral pleural invasion; NLNS: number of lymph nodes station.			

### 1.2 术后随访

随访通过门诊、住院复查和电话等形式完成。无病生存期（disease free survival, DFS）按月计算，以手术日期为观察起始点，终点事件为肿瘤复发或死亡；复发时间以初次（病理组织学检查）确定复发病灶为准。总生存期（overall survival, OS）指自观察起始点至死亡或末次随访。中位随访时间为36.0个月。截至2015年3月31日，随访内出现复发患者47例（18.2%），15例死亡（5.8%）。

### 1.3 统计学方法

所有数据采用SPSS 20.0软件进行统计学分析。计量资料用Mean±SD表示，计数资料用百分比表示。*Kaplan*-*Meier*法（*Log*-*rank*检验）比较生存曲线及统计学差异。用*Kaplan*-*Meier*法单因素分析得出有意义的临床病理因素纳入*Cox*回归模型进行多因素分析。采用*PSM*法对VATS肺叶切除和亚肺叶切除两组进行配对。协变量的选择是将结局变量与混杂因素构建*Logistic*回归模型进行逐步回归，进入模型的变量，包括年龄、性别、体重指数（body mass index, BMI）、肿瘤位置、肿瘤大小。利用最邻配比法从肺叶切除组中找出1个与亚肺叶切除组个体倾向评分最相近的个体进行配对^[7]^。检验水准：*α*=0.05，*P*＜0.05有统计学意义。

## 2 结果

VATS肺叶切除组和亚肺叶切除组术后DFS分别为56.8个月和42.9个月（*P*=0.016）；术后OS分别为65.8个月和49.1个月（*P*=0.003）（图 1），两者差异有统计学意义。单因素分析结果表明BMI、肿瘤大小、VPI、清扫淋巴结站数对术后DFS有显著影响，年龄、BMI、手术方式对术后OS有显著影响（表 2）。*Cox*多因素分析结果表明BMI和清扫淋巴结站数为术后DFS的独立预后风险因素，肿瘤大小和VATS术式为术后OS的独立预后风险因素（表 3）。

**1:**
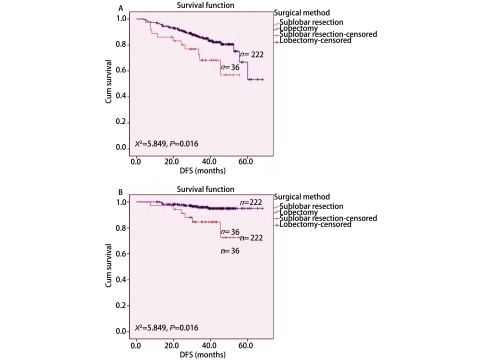
两种VATS术式治疗Ⅰ期肺腺癌患者的无病生存期曲线和总生存期曲线。A：无病生存期曲线；B：总生存期曲线。 DFS and OS curves of patients with stage Ⅰ lung adenocarcinoma after two kinds of VATS resection. A: DFS cueves; B: OS curves; VATS: video-assisted thoracoscopic surgery.

**2:** Ⅰ期肺腺癌患者的*Kaplan*-*Meier*法单因素分析 Univariate analysis of stageⅠ lung adenocarcinoma by *Kaplan*-*Meier* method

Variables	DFS			OS	
	*χ*^2^	*P*		*χ*^2^	*P*
Gender	2.319	0.128		0.049	0.824
Age (yr)	3.689	0.297		73.552	0.017
BMI (kg/m^2^)	21.650	＜0.001		14.991	0.005
Symptom	2.393	0.122		3.028	0.082
Smoking history	0.214	0.643		0.661	0.461
Tumor site	9.851	0.080		4.375	0.499
Tumor size (cm)	16.337	＜0.001		5.857	0.053
Surgical method	0.841	0.359		4.291	0.038
VPI	4.522	0.033		1.025	0.311
NLNS	37.461	＜0.001		2.732	0.950
DFS: disease free survival; OS: overall survival.					

**3:** Ⅰ期肺腺癌患者的*Cox*回归模型多因素分析 *Cox* regression model analysis of the stage Ⅰ lung adenocarcinoma patients

Variables	DFS				OS		
	HR	95%CI for HR	*P*		HR	95% CI for HR	*P*
Gender	0.730	0.376-1.149	0.354		1.756	0.546-5.649	0.345
Age (yr)	1.019	0.989-1.049	0.218		1.033	0.979-1.090	0.233
BMI (kg/m^2^)	0.935	0.890-0.983	0.009		0.960	0.871-1.057	0.405
Symptom	1.227	0.665-2.267	0.513		2.023	0.651-6.288	0.223
Smoking history	0.566	0.282-1.138	0.110		0.563	0.178-1.779	0.328
Tumor site	0.877	0.715-1.077	0.210		0.723	0.489-1.070	0.105
Tumor size (cm)	1.335	0.982-1.814	0.065		2.105	1.156-3.834	0.015
Surgical method	0.612	0.275-1.363	0.230		0.142	0.036-0.559	0.005
VPI	1.292	0.700-2.384	0.413		0.853	0.266-2.740	0.790
NLNS	0.797	0.655-0.970	0.024		0.938	0.657-1.340	0.725

通过*PSM*进行1:1两组匹配共35对（表 4）。本次PSM整体均衡性检验*P*=0.839，匹配后的L1统计量为0.857小于匹配前0.986，PS分布直方图（图 2）均提示匹配优良。VATS肺叶切除组与亚肺叶切除组的术后DFS分别为49.3个月和42.7个月，术后OS分别为50.3个月和49.0个月；两者差异均无统计学意义（图 3）。根据分期分层进行分析，Ia期肺叶切除和亚肺叶切除两组术后DFS分别为47.2个月和45.4个月，*P*=0.822；Ib期两组术后DFS分别为48.8个月和36.9个月（*P*=0.042）（图 4）。Ia期肺叶切除和亚肺叶切除两组术后OS分别为49.2个月和45.2个月，*P*=0.706；Ib期两组术后OS分别为49.7个月和47.9个月（*P*=0.088）（图 5）。

**4:** 倾向评分匹配后70例Ⅰ期肺腺癌患者的临床特征 Clinical characteristics of 70 patients with stage Ⅰ lung adenocarcinoma after PSM

Variable	Surgical method		*P* value
	Lobectomy (*n*=35)	Sublobectomy (*n*=35)	
Gender			0.810
Male	20 (57.1%)	21 (60.0%)	
Female	15 (42.9%)	14 (40.0%)	
Age (yr)	61±10	61±13	0.756
BMI (kg/m^2^)	23.33±3.40	22.13±4.89	0.240
Tumor size (cm)	2.2±1.0	2.0±1.0	0.456
Smoking status			0.800
Never	23 (65.7%)	23 (74.1%)	
Ever	11 (31.4%)	8 (6.7%)	
Unknown	1 (2.9%)	4 (9.3%)	
Tumor site			0.859
RUL	13 (37.1%)	13 (37.1%)	
RML	1 (2.9%)	0 (0.0)	
RLL	5 (14.3%)	6 (17.2%)	
LUL	11 (31.4%)	12 (34.3%)	
LLL	5 (14.3%)	4 (11.4%)	
PSM: propensity score matching.			

**2:**
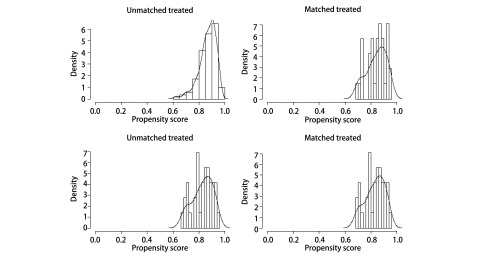
倾向评分分布直方图 Distribution of propensity scores of VATS sublobar resection group and lobectomy group before and after matching. VATS: video-assisted thoracoscopic surgery.

**3:**
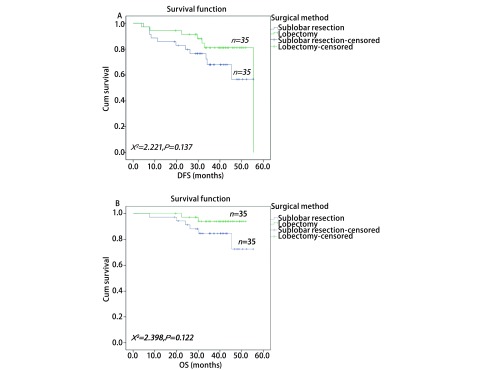
两种VATS术式治疗Ⅰ期肺腺癌患者的无病生存期曲线和总生存期曲线。A：无病生存期曲线；B：总生存期曲线。 DFS and OS curves of patients with stage Ⅰ lung adenocarcinoma after two kinds of VATS resection. A: DFS curves; B: OS curves.

**4:**
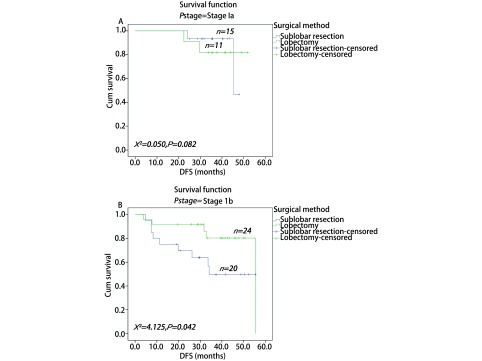
两种VATS术式治疗Ia期和Ib期肺腺癌患者的无病生存期曲线。A：Ia期；B：Ib期。 DFS curves of patients with stage Ia lung adenocarcinoma after two kinds of VATS resection. A: stage Ia; B: stage Ib.

**5:**
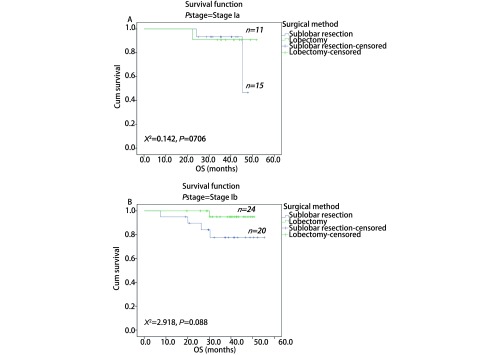
两种VATS术式治疗Ia和Ib期肺腺癌患者的总生存期曲线。A：Ia期；B：Ib期。 OS curves of patients with stage Ia and Ib lung adenocarcinoma after two kinds of VATS resection. A: stage Ia; B: stage Ib.

## 3 讨论

本研究通过对VATS肺叶切除和亚肺叶切除Ⅰ期肺腺癌两组患者进行倾向评分匹配，比较其远期生存预后。结果显示Ⅰ期肺腺癌VATS肺叶切除组患者的术后无病生存期和总生存期均大于亚肺叶切除组。分层分析后结果表明Ia期肺腺癌亚肺叶切除疗效不亚于肺叶切除，而肺叶切除Ib期患者的预后优于亚肺叶切除。

2014年Dembitzer等^[8]^在Chest杂志上报道了一项85例Ⅰ期肺腺癌的试点研究，结果肺叶切除组（*n*=59）与亚肺叶切除组（*n*=26）两者的生存预后差异无统计学意义。El-Sherif等^[9]^研究与上述Ⅰ期研究结果类似，接受亚肺叶切除（207例）和肺叶切除组（577例）治疗术后DFS的风险比（hazard ratio, HR）为1.20（95%CI: 0.90-1.61; *P*=0.240），两组患者术后OS的HR=1.39（95%CI: 1.11-1.75; *P*=0.004）。而Kates等^[10]^在≤1 cm的2, 090例Ⅰ期NSCLC患者人群中得到了类似结论，亚肺叶切除生存预后可达肺叶切除相同的远期疗效。

Nakamura等^[11]^通过Medline检索出14项研究共纳入肺叶切除1, 887例、亚肺叶切除903例Ⅰ期NSCLC患者进行荟萃分析，根据*DerSimonian*-*Laird*随机效应模型对两组的1年、3年、5年生存率差（肺叶切除组生存率减去亚肺叶切除组生存率）进行比较，结果分别为0.7%、1.9%、3.6%，三者均无统计学意义。然而，2015年Zhang等^[12]^报道的荟萃分析结果显示，Ⅰ期NSCLC肺段切除与肺叶切除的HR=1.231（95%CI: 1.070-1.417, *P*=0.004），肺段切除比肺叶切除更适合治疗Ia期NSCLC且可获得同等疗效。

尽管LCSG研究已证实外周型T1N0的NSCLC局限性切除的预后差^[2]^。但2013年Tsutani等^[13]^研究却证实肺段切除Ia期肺腺癌的3年无瘤生存期（relapse relapse-free survival, RFS）和OS与肺叶切除结果均无明显差异。2014年，Okada等^[14]^通过研究高分辨率CT（High Resolution CT, HRCT）或PET/CT显示以磨玻璃影（ground glass opacity, GGO）为主610例临床分期为Ia期的肺腺癌，肺叶切除、肺段切除和肺楔形切除术后3年的RFS分别为96.4%、96.1%和98.7%（*P*=0.440），考虑到T1b肺腺癌中2.4%（2/84）患者有淋巴结转移，因此该研究得出结论T1a肿瘤可适合行肺楔形切除，T1b可行肺段切除。最近Koike等^[15]^在251例影像学显示纯实性T1aN0M0期NSCLC中也得出肺段切除术后10年的DFS和OS与肺叶切除无显著差异。

此外，Okada等^[16]^提出建议对外周型≤2 cm的Ia期NSCLC可考虑行亚肺叶切除，该观点得到了包括荟萃分析在内的多项研究结果的证实^[17-20]^。然而，2015年美国临床肿瘤学杂志发表了Veluswamy等^[21]^通过SEER数据库回顾性分析65岁以上且≤2 cm的Ia期NSCLC的研究，结果却证实肺叶切除组预后更佳。Chamogeorgakis等^[22]^研究结果的观点与Veluswamy等研究^[21]^相似，即Ia期肺癌仍推荐行标准解剖性肺叶切除^[23]^。而针对Ib期患者，本研究经匹配后肺叶切除的DFS和OS预后均优于亚肺叶切除（*P*＜0.05）。该原因可能与部分Ib期患者术后接受化疗等因素相关。目前，有学者开展亚肺叶切除和肺叶切除治疗早期NSCLC预后对比的临床试验研究^[24]^。

虽然本研究对年龄、性别、BMI、肿瘤位置、肿瘤大小等因素进行了倾向评分匹配分析，可在一定程度控制病例的选择性偏倚。然而，本研究仍存在以下局限性：①回顾性分析的研究性质；②纳入VATS亚肺叶切除治疗的肺腺癌患者均为经验丰富的临床胸外科专家筛选，会产生研究上的偏倚。本研究纳入的Ⅰ期肺腺癌患者病例数量偏少、中位随访时间较短。

总之，本研究通过倾向评分匹配后对比肺叶切除和亚肺叶切除治疗预后，显示Ia期肺腺癌VATS亚肺叶切除治疗预后不比肺叶切除差。针对Ib期肺腺癌患者，我们仍建议首选VATS解剖性肺叶切除治疗。然而该研究只是我院单中心的回顾性研究分析，其结果的科学性和普遍性还需未来多中心、大样本及前瞻性的临床研究来进一步证实完善。
